# Letter of A. Berry

**Published:** 1852-01

**Authors:** 


Raymond, Miss., August 31, 1851.

Dr. Taylor—Dear Sir: I received yours some time ago, in which you remind me of my duty to assist in preparing the report for Dr. Drake, I regret very much that the tables of my examinations of the teeth of some four or five hundred negroes, (field hands,) from the age of fifteen years upward, were left at Cincinnati last fall. I intended to copy from them for the report, but as I cannot do this, I don't think any portion I could write would be of much utility in preparing it.

I will merely make a few remarks on the subject. The questions, or part of them, are not fairly put, as there are no negroes, as a class, living in a similar manner with whites. I have not the questions with me.

From my observations, I am convinced that the teeth of the negroes are fully as liable to be attacked with caries as those of the whites living in a similar manner.

Their front teeth, I think, decay less, but their posterior teeth more than those of the whites. Their teeth seem not to be so well organized, owing, probably to their food—indian corn and swine's flesh—being deficient in this ingredient. None of them, as you know, ever brush or cleanse their teeth in any way.

Among those examined, I found about five in a hundred over fifteen years with a sound set of teeth; those youngest usually having their anterior molars carious.*

Respectfully yours, A. Berry.

---

*The above letter from Dr. Berry was received previous to the last meeting of the Mississippi Valley Association of Dental Surgeons, and we intended to publish it with the synopsis of the discussions on Dr. Drake's interrogatories, but from some cause or other it was omitted. We are aware that it was not designed for publication, yet as it embraces very much our own views on the subject of the decay of the teeth among the blacks in the South, we hope the doctor will excuse us for its publication. We presume, however, that Dr. Drake's question is only designed to apply to that class of whites who are obliged to live on simple diet, and whose habits of cleanliness are much the same as the blacks. We have noticed the more frequent loss of the molar teeth among the blacks, and the fact that the front teeth are not so often lost. We wonld ask, in this connection, one question: Are their teeth so apt to be crowded? One of the most striking physiognomic characteristics of the African race, is a flattened face, which gives an expanded arch to the front teeth.—Ed.

